# Swiss Cohort & Biobank—An Ambitious and Timely Large-Scale Cohort Study

**DOI:** 10.3389/ijph.2023.1605886

**Published:** 2023-03-17

**Authors:** Annette Peters

**Affiliations:** ^1^ Institute of Epidemiology, Helmholtz Zentrum München, German Research Center for Environmental Health (GmbH), Neuherberg, Germany; ^2^ Chair of Epidemiology, Institute for Medical Information Processing, Biometry and Epidemiology, Medical Faculty, Ludwig-Maximilians-Universität München, Munich, Germany; ^3^ Munich Heart Alliance, German Center for Cardiovascular Research (DZHK e.V., Partner-Site Munich), Munich, Germany; ^4^ German Center for Diabetes Research (DZD e.V.), Neuherberg, Germany; ^5^ Department of Environmental Health, Harvard T. H. Chan School of Public Health, Boston, MA, United States

**Keywords:** cohort studies, public health, non-communicable diseases, infectious diseases, Exposome

Over time, life expectancy has increased and disease patterns have shifted globally (1, 2). This major success is attributable to public health interventions, as well as substantial improvements in and accessibility of healthcare. Our understanding of risk factors for early onset and progression of non-communicable diseases has improved substantially. Within the last century, population-based research established the role of lifestyle determinants of disease including smoking, low physical activity, and nutrition. Twenty-first century research has made major breakthroughs by characterizing the genetic contribution to diseases, establishing polygenic risk scores, and quantifying environmental exposures such as air pollution as major contributors to the global burden of disease. Nevertheless, the COVID-19 pandemic showed that we were ill prepared for dealing with novel emerging infectious diseases. Now in the aftermath of the COVID-19 pandemic, we realize the imminent need for high quality, prospective, population-based cohort data and public health surveillance of non-communicable and infectious diseases (3).

Europe has a longstanding tradition of successfully implementing and maintaining population-based cohorts over decades. Large-scale cohort studies, in particular studies with more than 100,000 participants, enable researchers to address the complexity of risk and protective factors jointly, to understand underlying joint disease mechanisms, and to assess the incidence of complex diseases and their subtypes prospectively. Furthermore, they allow for the assessment of the effectiveness of health promotion and primary prevention for the decades to come. Establishing a large-scale cohort is an enormous and multi-disciplinary endeavor and requires substantial planning that builds upon experience. Large-scale cohort studies such as, for example, the UK Biobank (4), Lifelines (5), Constances (6), or the German National Cohort (NAKO) (7) showed that it is feasible to recruit and characterize large population groups (Figure 1).

**FIGURE 1:**
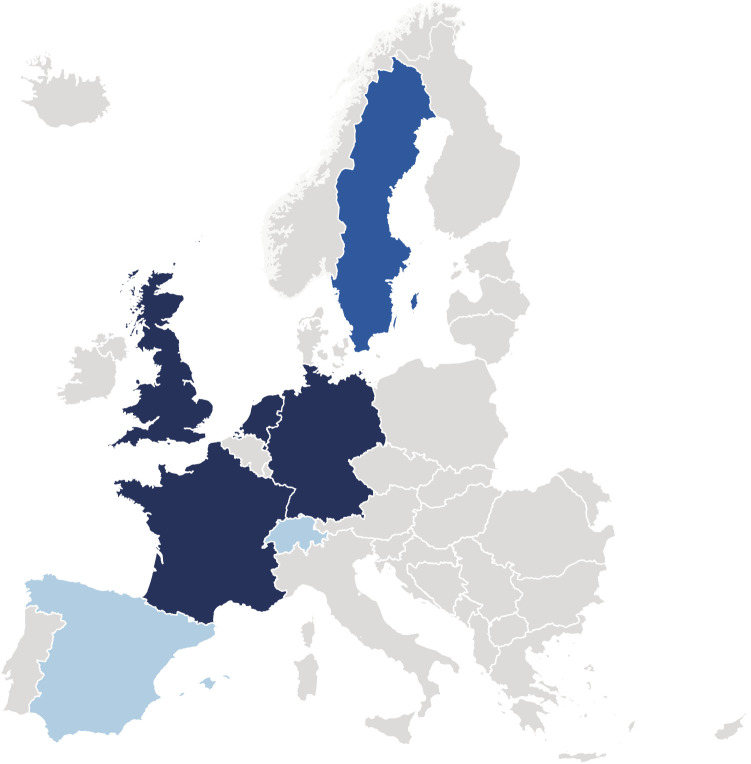
Prospective cohort study with at least 100,000 participants including examinations, biobanking, and follow-up for incident disease, founded after 2000: British UK Biobank, Dutch Lifelines, French Constances, German National Cohort (NAKO) (dark blue). Ongoing recruitments: Swedish Lifegene (blue). Planned large-scale cohorts: Swiss Cohort & Biobank and Spanish National Cohort (light blue). © EuroGeographics for the administrative boundaries.

The concept for the Swiss Cohort & Biobank described in the white paper by Probst-Hensch et al. (3) puts forward a timely and ambitious concept. The Swiss Cohort & Biobank is designed to address specific cultural, ethnic, social, and environmental circumstances to optimally promote health and tailor efforts towards the Swiss healthcare system. The concept builds upon ongoing cohort studies in Switzerland and proposes important innovation, while building upon successful large-scale cohorts within Europe to ensure comparability and interoperability of data. The innovations include recruitment from birth to old age at baseline, implementation of novel eHealth tools, and citizens’ involvement during planning and execution to overcome difficulties in response rates. The scientists spearheading the efforts represent major academic institutions, universities, and societies of public health and have all the needed scientific experiences to embark on such a challenging endeavor. It will be important to ensure long-term sustainable funding for study centers, biobanking, and central digital infrastructures for data storage and data access. The vision is to enrich the core study of 100,000 participants with disease-specific cohorts, deeply phenotyped sub-cohorts, and family members. From an ethical standpoint, it is important to protect the individuals who provide data and biosamples, so that their personal rights and access to healthcare, insurances, and labor markets are not jeopardized. From a public health standpoint, it is equally important to link to other data sources to enrich cohort databases with high quality data stored within the healthcare system, from public authorities and from other databases with relevance for health. Therefore, an endorsement on a broad societal and institutional level is needed.

The Swiss Cohort & Biobank has the ambition to collect biomaterials based on state-of-the-art approaches to allow for sequencing and in-depth molecular characterization. These biosamples and data will allow the promotion of biomedical research and the integration of genome and exposome concepts (8). Taken together, the Swiss Cohort & Biobank will advance the understanding of causality in disease etiology. Novel technologies are envisioned to be used for environmental characterization, molecular phenotyping, or imaging. These approaches together will allow developing fingerprints of preclinical and clinical disease and allow implementing holistic innovative concepts such as linking the hallmarks of environmental insults to disease (9). The concept of the Swiss Cohort & Biobank does not stop there, but explicitly calls to integrate psychological and sociological expertise. Taken together, the Swiss Cohort & Biobank is suited to transform public health and biomedical research in Switzerland and internationally.

Recently, Fagherazzi called for a “cohort moonshot” (10) that would allow sustained and long-term follow-ups within Europe (Figure 1). Indeed, challenges such as population growth and ageing societies, urbanization, globalization and supply chain dependencies, digital transformation, increasing social disparities, the climate crisis and biodiversity loss, as well as military conflicts will impact public health globally as well as locally (3, 7). Large-scale cohorts are a necessary investment into understanding the challenges of tomorrow. They provide evidence where randomized trials are unethical or unfeasible (7). They are planned serendipity and enable the realization of that which is unconceivable today.

In conclusion, large-scale cohort studies with the Swiss Cohort & Biobank as a prime example are ideal tools to derive public health interventions and to evaluate policies considering individual and population heterogeneities. Such investments will provide effective and cost-effective approaches to promote primary prevention at the population level.

## References

[1] GBD 2019 Diseases and Injuries Collaborators. Global burden of 369 Diseases and Injuries in 204 Countries and Territories, 1990-2019: a Systematic Analysis for the Global Burden of Disease Study 2019. Lancet (2020) 396(10258):1204–22. 10.1016/S0140-6736(20)30925-9 33069326 PMC7567026

[2] GBD 2019 Demographics Collaborators. Global Age-sex-specific Fertility, Mortality, Healthy Life Expectancy (HALE), and Population Estimates in 204 Countries and Territories, 1950-2019: a Comprehensive Demographic Analysis for the Global Burden of Disease Study 2019. Lancet (2020) 396(10258):1160–203. 10.1016/S0140-6736(20)30977-6 33069325 PMC7566045

[3] Probst-Hensch N Bochud M Chiolero A Crivelli L Dratva J Flahault A Swiss Cohort & Biobank - the White Paper. Public Health Rev (2022) 43:1605660. 10.3389/phrs.2022.1605660 36619237 PMC9817110

[4] Sudlow C Gallacher J Allen N Beral V Burton P Danesh J UK Biobank: an Open Access Resource for Identifying the Causes of a Wide Range of Complex Diseases of Middle and Old Age. Plos Med (2015) 12(3):e1001779. 10.1371/journal.pmed.1001779 25826379 PMC4380465

[5] Stolk RP Rosmalen JG Postma DS de Boer RA Navis G Slaets JP Universal Risk Factors for Multifactorial Diseases: LifeLines: a Three-Generation Population-Based Study. Eur J Epidemiol (2008) 23(1):67–74. 10.1007/s10654-007-9204-4 18075776

[6] Zins M Bonenfant S Carton M Coeuret-Pellicer M Gueguen A Gourmelen J The CONSTANCES Cohort: an Open Epidemiological Laboratory. BMC Public Health (2010) 10:479. 10.1186/1471-2458-10-479 20704723 PMC2927544

[7] Peters A German National Cohort C Peters A Greiser KH Gottlicher S Ahrens W Framework and Baseline Examination of the German National Cohort (NAKO). Eur J Epidemiol (2022) 37(10):1107–24. 10.1007/s10654-022-00890-5 36260190 PMC9581448

[8] Vlaanderen J de Hoogh K Hoek G Peters A Probst-Hensch N Scalbert A Developing the Building Blocks to Elucidate the Impact of the Urban Exposome on Cardiometabolic-Pulmonary Disease: The EU EXPANSE Project. Environ Epidemiol (2021) 5(4):e162. 10.1097/EE9.0000000000000162 34414346 PMC8367039

[9] Peters A Nawrot TS Baccarelli AA. Hallmarks of Environmental Insults. Cell (2021) 184(6):1455–68. 10.1016/j.cell.2021.01.043 33657411 PMC9396710

[10] Fagherazzi G. Towards a European "Cohort Moonshot": Revisiting the Long-Term Strategy to Support Health Research of Tomorrow. Eur J Epidemiol (2023) 38(1):121–2. 10.1007/s10654-022-00939-5 36598706 PMC9867653

